# The Grapevine and Wine Microbiome: Insights from High-Throughput Amplicon Sequencing

**DOI:** 10.3389/fmicb.2017.00820

**Published:** 2017-05-11

**Authors:** Horatio H. Morgan, Maret du Toit, Mathabatha E. Setati

**Affiliations:** Department of Viticulture and Oenology, Institute for Wine Biotechnology, Stellenbosch UniversityStellenbosch, South Africa

**Keywords:** amplicon sequencing, vineyard microbiome, microbial diversity, wine fermentation, non-*Saccharomyces* yeasts

## Abstract

From the time when microbial activity in wine fermentation was first demonstrated, the microbial ecology of the vineyard, grape, and wine has been extensively investigated using culture-based methods. However, the last 2 decades have been characterized by an important change in the approaches used for microbial examination, due to the introduction of DNA-based community fingerprinting methods such as DGGE, SSCP, T-RFLP, and ARISA. These approaches allowed for the exploration of microbial community structures without the need to cultivate, and have been extensively applied to decipher the microbial populations associated with the grapevine as well as the microbial dynamics throughout grape berry ripening and wine fermentation. These techniques are well-established for the rapid more sensitive profiling of microbial communities; however, they often do not provide direct taxonomic information and possess limited ability to detect the presence of rare taxa and taxa with low abundance. Consequently, the past 5 years have seen an upsurge in the application of high-throughput sequencing methods for the in-depth assessment of the grapevine and wine microbiome. Although a relatively new approach in wine sciences, these methods reveal a considerably greater diversity than previously reported, and identified several species that had not yet been reported. The aim of the current review is to highlight the contribution of high-throughput next generation sequencing and metagenomics approaches to vineyard microbial ecology especially unraveling the influence of vineyard management practices on microbial diversity.

## Introduction

The conversion of grape juice into wine was first confirmed to be the result of a microbial process by Louis Pasteur in the middle of the nineteenth-century (Barnett, 2003; Jolly et al., 2014; Bokulich et al., 2016b). Since then, the diversity of the vineyard, grape and wine microbiota has been extensively investigated using traditional microbiological methods involving microscopy, cultivation on different agar media and biochemical characteristics. However, the arrival of DNA-based molecular techniques such as polymerase chain reaction (PCR) and the identification of evolutionarily stable molecular marker genes such as ribosomal RNA (rRNA) genes improved our ability to identify microbial species with better resolution and reliability (Justé et al., 2008; Solieri and Giudici, 2008; Cocolin et al., 2013; Sun and Liu, 2014; Wang et al., 2014; Abbasian et al., 2015b). The bacterial small subunit ribosomal RNA gene (16S rRNA) as well as the fungal ITS1-5.8S rRNA-ITS2 gene have been recognized as the gold standard for estimating the phylogenetic diversity in microbial communities (Justé et al., 2008; Cocolin et al., 2013; Sun and Liu, 2014). Consequently, for the past 3 decades, molecular techniques relying on rRNA genes as target molecules, have been employed in conjunction with culture-dependent methodologies to identify microorganisms after isolation and growth in pure cultures (Esteve-Zarzoso, 1999; Alessandria et al., 2013; Cocolin et al., 2013). To date more than 40 yeast species (Jolly et al., 2014), 50 bacterial species (Barata et al., 2012) and ~70 genera of filamentous fungi (Rousseaux et al., 2014) associated with grapevine and wine fermentation processes have been isolated and identified using traditional culture-based methods. These methods are however extremely laborious, time consuming and often inconsistent and biased (Andorrà et al., 2008; Sun and Liu, 2014). In addition, only species that are able to grow on the culture media and under the cultivation conditions used can be isolated and identified, while species that are in low abundance, those species for which the prevailing cultivation conditions are not conducive, as well as viable but non-culturable (VBNC) cells, are often overlooked (Abbasian et al., 2015b). These limitations in culture-based methods as well as the difference between culturable and *in situ* diversity increased the importance for research into culture-independent molecular approaches (Nocker et al., 2007). Nevertheless, these methods remain important since the microbial species and strains retrieved in such culture-based approaches can be further exploited depending on their biochemical or genetic profiles. Indeed, the wine industry today has access to more than 100 commercial active dry yeast (ADY) strains of *Saccharomyces cerevisiae* that are used as starter cultures for controlled fermentations (Fernández-Espinar et al., 2001; Guzzon et al., 2014). More recently, strains of non-*Saccharomyces* yeasts such as *Torulaspora delbrueckii, Metschnikowia pulcherrima, Lachancea thermotolerans*, and *Pichia kluyveri*, and several others have been made available as pure starter cultures and in blends with *S. cerevisiae* (Lu et al., 2016; Padilla et al., 2016).

Introduction of PCR-based methods further created opportunities for the development and improvement of several techniques in molecular ecology. The application of molecular techniques allowed researchers to study microbes not on the basis of their ability to grow on certain media types but rather relied on the presence nucleic acids for detection and identification. Such methods, mostly use DNA extracted directly from the environment as a template for PCR, followed by separation and detection for microbial community profiling. Culture-independent methods are often faster, more sensitive and have a higher accuracy than culture-dependent methods (Justé et al., 2008; Lv et al., 2013). These methods include, single-strand conformational polymorphisms (SSCP), denaturing gradient gel electrophoresis (DGGE), terminal restriction fragment length polymorphisms (T-RFLP), and automated ribosomal intergenic spacer analysis (ARISA; Justé et al., 2008; Kovacs et al., 2010; Slabbert et al., 2010; Balázs et al., 2013; Cocolin et al., 2013; Abbasian et al., 2015b). PCR-DGGE was first applied in wine fermentation by Cocolin et al. (2001) to monitor the diversity and dynamics of yeast populations. Since then, it has remained the most widely used community profiling method in wine fermentation, also including bacteria (Renouf et al., 2006a,b; Cameron et al., 2013). The technique is often employed in combination with culture-dependent methods and has allowed researchers to decipher the complexity and evolution of the microbial population, during berry ripening and throughout the fermentation process (Prakitchaiwattana et al., 2004; Renouf et al., 2005, 2007; Di Maro et al., 2007; Andorrà et al., 2008). Although PCR-DGGE is typically thought to be appropriate for the analysis of less species-rich environments such as grape must, it has low sensitivity (Andorrà et al., 2010) and is unable to detect populations that are present at a relative abundance of <1% of the population (Fasoli et al., 2003; Andorrà et al., 2008). More recently, SSCP (Grube et al., 2011; Schmid et al., 2011; Martins et al., 2014), T-RFLP (Martins et al., 2012; Sun and Liu, 2014), and ARISA (Brežná et al., 2010; Chovanová et al., 2011; Kraková et al., 2012; Pancher et al., 2012; Setati et al., 2012; Ženišová et al., 2014; Ghosh et al., 2015) have been employed to profile the wine microbial diversity. Culture-independent methods also allow researchers to monitor populations that are numerically under-represented as well as those in the VBNC state (Andorrà et al., 2010; Cocolin et al., 2013). It is critical to monitor such populations as they can influence wine quality. For instance, several studies have demonstrated that strains of *S. cerevisiae, Zygosaccharomyces baillii*, and *Brettanomyces bruxellensis* when exposed to SO_2_ can enter into VBNC state and survive for more than a month depending on the pH of the environment (Divol and Lonvaud-Funel, 2005; Salma et al., 2013; Capozzi et al., 2016). During this state a spoilage yeast such as *B. bruxellensis* can produce volatile phenols that impart off-odors to the final wine thus rendering it unpalatable (Salma et al., 2013; Capozzi et al., 2016). Although the culture-independent methods have allowed researchers to detect and monitor the evolution of microbial communities, and capture species that were previously not detected, or even misrepresented with culture-dependent methods (Peršoh, 2015), they do have several limitations associated with each of the methods (Table 1). Such limitations, e.g., poor band-resolution, co-migration of species, and PCR amplification biases mean that diversity analysis based on these methods still provides a narrow view of the community composition.

**Table 1:** **A summary of the advantages and disadvantages of PCR-based culture-independent microbial community fingerprinting methods (Arteau et al., 2010; Cocolin et al., 2013)**.

**Methods**	**Advantages**	**Disadvantages**
Single-strand conformational polymorphisms (SSCP)	• Distinct bands can be isolated and sequenced • No clamped primers and REs required	• High rate of re-annealing of single strands with high DNA concentrations
Denaturing gradient gel electrophoresis (DGGE)	• Ability to target both RNA and DNA	• Only intense and well-separated bands can be sequenced
Real-time quantitative PCR (QPCR)	• Can be applied to RNA and therefore measures viable population	• Abundance quantification may be affected by differences in gene expression at different physiological state of the cells • Requires species specific primers
Terminal restriction fragment length polymorphisms (T-RFLP)	• Easily applicable to large sample numbers • Web-based tools allow *in silico* prediction of TRFs	• Incomplete and non-specific digestion leads to overestimation of diversity • Poor resolution of complex communities • Requires multiple RE's
Automated ribosomal intergenic spacer analysis (ARISA)	• Less labor intensive • Allows detection of dominant species • Allows high resolution of subtle differences	• Co-migration of species with same ITS amplicon size • Preferential amplification of shorter templates

Improvements in DNA sequencing, expanded the ability of researchers to study the microbial community structure and function with a higher resolution by employing metagenomic approaches. Metagenomics can be defined as the direct genetic analysis of the collective of genomes within an environmental sample (Thomas et al., 2012), this can be achieved either through whole metagenome sequencing or amplicon-based sequencing. Amplicon sequencing, often grouped under the umbrella of metagenomics, is a culture-independent approach for taxonomic, phylogenetic, or functional profiling of microbial communities, accomplished by sequencing specific marker genes amplified directly from environmental DNA without prior enrichment or cultivation of the target population (Franzosa et al., 2015). The innovations in high-throughput, short-amplicon sequencing are revolutionary in a way that they can describe the microbial diversity within and across complex biomes (Bokulich et al., 2013b). Although high-throughput sequencing technologies have been widely used to investigate the microbial ecology of various environments (Ma et al., 2015; Shi et al., 2015; Abbasian et al., 2015a), their application in grapevine and wine fermentation microbial ecology is relatively recent, and their contribution to the field has not been explored. In recent studies it was also shown that grape microbial diversity is driven by cultivar, climatic conditions both macro- and micro-climate, the seasonal environmental conditions, viticultural farming practices as well as wine microbiome by fermentation process applied during the winemaking (Bokulich et al., 2014; David et al., 2014; Gilbert et al., 2014; Setati et al., 2015; Zarraonaindia et al., 2015; Abbasian et al., 2015a; De Filippis et al., 2017). Therefore, with this review, we aim to provide an in-depth overview of the vineyard, grape, and wine microbiome and its functional potential as unraveled through high-throughput sequencing techniques.

## Next-generation sequencing

For many years, microbial community analyses relied on the isolation and identification of individual species, or cloning and sequencing of rRNA genes retrieved by PCR from environmental DNA. These methods mainly relied on first-generation DNA sequencing technology which was developed by Sanger et al. (1977). A few decades later, deep high-throughput, in-parallel sequencing technologies collectively referred to as Next-generation sequencing (NGS) were developed (Bleidorn, 2015). The term NGS therefore specifically refers to non-Sanger-based second and third generation sequencing (TGS) techniques (Türktaş et al., 2015).

After Sanger introduced the chain-terminator DNA sequencing method, commercial second generation sequencing (SGS) platforms were developed. The Genome Sequencer 20 system launched in 2005 by 454 Life Sciences, was the first commercial SGS platform and was soon followed by the Genome Analyzer II launched by Solexa/Illumina in 2006. Both these platforms use a sequencing by synthesis approach. Roughly 2 years later, Lifetechnologies/Applied Biosystems introduced the SOLiD (Sequencing by Oligonucleotide Ligation and Detection) platform which applies fluorophore labeled oligonucleotide panels and ligation chemistry for sequencing. Subsequently, Complete Genomics developed the CGA sequencing technology which employed semi-ordered array of “DNA nanoballs” on a solid surface, while the Ion Torrent, which is regarded as the first of the “post-light sequencing” technologies, was introduced in 2010 (Reuter et al., 2015; Heather and Chain, 2016). The Ion Torrent's semiconductor sequencer is thought to be a technology between second and TGS categories. The technology is capable of sequencing single molecules thus negating the requirement for prior DNA amplification (Heather and Chain, 2016).

The majority of SGS technologies however, still have various limitations, such as errors arising from PCR (Peršoh, 2015), the loss of synchronicity “dephasing” (Schadt et al., 2010; Diaz-Sanchez et al., 2013) and the duration of completion “time to results” (Diaz-Sanchez et al., 2013). To overcome these drawbacks TGS or next-next generation platforms such as Single-molecule real-time (SMRT) sequencing (Schadt et al., 2010; Bleidorn, 2015) and Nanopore DNA Sequencer (Diaz-Sanchez et al., 2013), which open the possibility for single molecule sequencing were developed. These come with several advantages, (i) higher throughput, (ii) faster “time-to-result,” (iii) low cost, (iv) longer read length, (v) increased consensus accuracy enabling rare variant detection and (vi) small starting material (Schadt et al., 2010; Diaz-Sanchez et al., 2013; Bleidorn, 2015). However, these sequencing methodologies are still in development, and/or in the beta stage. Few commercial platforms have been evaluated, however they remain plagued by high error rates, and low output, although the technology is promising (Bleidorn, 2015). As such they cannot yet replace SGS, which remain and continue to be pivotal in microbial ecology surveys.

## Next-generation sequencing in microbial ecology

SGS platforms have revolutionized the landscape of microbial ecology and have been the cornerstone of many phylogenetic surveys. The methods make it possible to compare and analyze the whole microbial community diversity, abundance, and functional genes at far greater sequencing depths. These technologies depend on a parallel process in which each single DNA fragment is sequenced independently and separated in clonal amplicons for downstream analysis between the total sequences generated (Wooley et al., 2010; Diaz-Sanchez et al., 2013). With most SGS methodologies, an uninterrupted operation of a washing and scanning process is used to read tens of thousands of matching strands that are fixed to a specific location (Schadt et al., 2010). The length of the fragments obtained from the analyses differs depending on the sequencing method employed (Wooley et al., 2010; Bokulich et al., 2016b). Until recently, the Illumina and 454 pyrosequencing platforms were the most commonly used platforms for grapevine ecology surveys. At least 48% of the published data on the vineyard, grapevine and wine microbiome is derived from 454 pyrosequencing while the remaining 52% is derived from Illumina sequencing. Both platforms work on a sequencing-by-synthesis approach, however differ in their chemistries.

## Illumina

The process of Illumina sequencing, consists of the bridge amplification of adapter-ligated DNA fragments on the surface of a glass (Pettersson et al., 2009). Bases are then determined using a cyclic reversible termination technique, which sequences the template strand, a single nucleotide at a time through progressive rounds of base incorporation, washing, scanning, and cleaning. In this method, labeled dNTPs are used to stop the polymerization reaction, allowing the removal of unincorporated bases. The fluorescent dye is captured to identify the bases added, and then cleaved so that the next nucleotide can be added, this is then repeated (Pettersson et al., 2009; Diaz-Sanchez et al., 2013; Reuter et al., 2015; Heather and Chain, 2016). The earlier Illumina analyser generated at least 1 Gb of sequences with reads averaging 35 bp and the duration of 2–3 days. However, the introduction of HiSeq and MiSeq machines altered the duration time to ~4 days and 24–30 h, and increased the read length to 250–300 bp, respectively with error rates of below 1%, with substitution the most occurring issue (Bleidorn, 2015; Goodwin et al., 2016).

## Pyrosequencing

In 454 pyrosequencing an emulsion PCR is used for bridge amplification of adapter-ligated DNA fragments on the surface of a bead. The beads are thereafter distributed and fixed into 44 μm wells, where the sequencing by synthesis occurs. After the nucleotide bases are incorporated an enzymatic luciferase coupled reaction occurs, allowing for the identification of bases, which is measured using a charged couple device (Pettersson et al., 2009; Diaz-Sanchez et al., 2013; Reuter et al., 2015; Heather and Chain, 2016). The Roche 454 FLX platform has the ability to generate 80–120 Mb of sequences averaging in 200–300 bp reads, for a run that averages ~4 h with an error rate of below 1% (Morozova and Marra, 2008), while the FLX titanium is capable of producing read lengths of over 400 bp (Pettersson et al., 2009).

The 454 pyrosequencing technique was reported in 2008, as the most published NGS platform, however, the technology has since been discontinued, and has therefore been surpassed by Illumina which is currently considered to have made the largest contribution to SGS (Huse et al., 2007; Morozova and Marra, 2008; Reuter et al., 2015; Heather and Chain, 2016).

## Application of next-generation sequencing in deciphering the vineyard microbiome

The vineyard microbiome broadly describes the collective genomes of microorganisms present in the vineyard ecosystem, including those present in soil, grapevine, cover crops, and the insects associated with the plants. Furthermore, microbial transfer from nearby plants, could be transported aerially or via insects (Gilbert et al., 2014). Consequently, the grape microbiome represents a reservoir of microorganisms comprising filamentous fungi, yeast as well as bacteria. These populations are however variable and are influenced by various external factors, such as grape cultivar, climatic conditions, farming practices, and the vineyard location (Setati et al., 2012; Salvetti et al., 2016). The past decade has seen a significant advancement in the manner in which researchers understand the microbial ecology of the vineyard, due to molecular profiling techniques that have further evolved, to explore microbial ecosystems (Bokulich et al., 2012). Recent studies have employed SGS to decipher the grape and grapevine associated microbiome (David et al., 2014; Pinto et al., 2014), and to determine how viticultural practices could potentially influence these communities (Setati et al., 2015; Kecskeméti et al., 2016; Marzano et al., 2016), their dynamics throughout grape berry development and wine fermentation (Piao et al., 2015; Stefanini et al., 2016) and to unravel their functional potential (Salvetti et al., 2016).

For the comprehensive evaluation of the vineyard and the grape microbiome, two key questions are typically addressed. Firstly, which microorganisms are present within the environment, and secondly the role of the individual species (Ravin et al., 2015). To understand what role the identified species, if any; plays in the grape and wine microbiome requires that standard microbiological methods be applied to isolate the strains and then evaluate them for their potential contribution to grape or wine quality by assessing their phenotypic and genotypic properties and thereafter they will be evaluated in different wine matrices to assess their growth and metabolic profile. To this effect, several species retrieved using culture-dependent methods have been shown to contribute positively in the winemaking process. For instance, some strains of *Wickerhamomyces anomalus, Candida pyralidae, T. delbrueckii*, and *Kluyveromyces wickerhamii* were shown to suppress the growth of *B. bruxellensis* (Comitini et al., 2017), a wine spoilage yeast; *M. pulcherrima* was highlighted as a desirable co-inoculant for the reduction of ethanol (Morales et al., 2015), while others such as *Hanseniaspora vineae, Starmerella bacillaris, L. thermotolerans, P. kluyveri*, and *T. delbrueckii* present various desirable aroma signatures (Jolly et al., 2014; Comitini et al., 2017). In order to explore the untapped diversity revealed by SGS, it would be important to establish enrichment methods that can allow retrieval of those species that have not yet been characterized. Consequently, different sampling strategies are employed depending on what question the researcher seek to address.

## Sampling strategies

The vineyard and grapevine microbiome has been studied from a variety of samples including the soil and different parts of the vines. However, there is currently no standardized sampling strategy or experimental design for vineyard microbiome analysis. For the soil microbiome samples are typically derived from surface soil or from the root zone. Typically, anything from 3 to 5 samples are randomly collected, sifted through a 0–2 mm sieve and then homogenized and composited. Samples are often collected with a spade or with the aid of a 33 inch by 7–8-inch corer, within the alleyways of the vineyard or at a distance of 15–30 cm away from the trunk, at a depth of 0–7 cm (Martins et al., 2014; Burns et al., 2015; Zarraonaindia et al., 2015). In contrast, root soil samples are collected closer to the stem (10–15 cm) although at similar depth to the surface samples (Zarraonaindia et al., 2015). For microbial evaluation of plant material such as roots and branches (Campisano et al., 2014), grapevines of similar age and size are typically chosen, eliminating one source of microbial variability. Only a certain area of the vine is sampled, the material typically peeled or crushed under aseptic conditions for further evaluation. For instance, some studies have used leaves (Leveau and Tech, 2011; Pinto et al., 2014) while others have used the cane, graft union of the trunk as well as the roots (Faist et al., 2016), depending on the aim of the study. In contrast, sampling for analysis of the grape-associated microbiome can vary from a few bunches to kilograms of grapes (David et al., 2014; Taylor et al., 2014; Pinto et al., 2015; Setati et al., 2015; Wang et al., 2015; Salvetti et al., 2016). Careful selection of healthy and undamaged grapes is often critical unless the aim is to investigate botrytized wines (Bokulich et al., 2012) and/or sweet wines (Stefanini et al., 2016). The grapes are subsequently crushed under asceptic conditions and the DNA extracted from the resulting must. In a few cases, samples were collected from commercial wineries as composite grape must (Bokulich et al., 2014, 2016a). In a few studies that monitored population dynamics during fermentation, additional samples are withdrawn at various time points representing the beginning, middle, and end of fermentation (David et al., 2014; Pinto et al., 2015; Wang et al., 2015). In most instances, sample volumes ranging from 5 to 50 mL are then further used for DNA extractions.

## Target genes

The target marker genes are universally present in all species evaluated and contain both highly conserved fragments that facilitate the design of PCR primers targeting all members of a community and variable regions that allow for the discrimination of different species within the community (Justé et al., 2008; Cocolin et al., 2013; Sun and Liu, 2014; Wang et al., 2014). In both fungi and bacteria, ribosomal RNA genes are suitable target genes. In bacteria, the 16S rRNA is typically targeted while in fungi the ITS1-5.8S rRNA-ITS2 as well as the 26S rRNA are the target molecules for high throughput amplicon sequencing and microbiome analyses.

The 9 hypervariable regions (V1–V9) of bacteria have all been targeted for the estimation of vineyard bacterial diversity (Leveau and Tech, 2011; Campisano et al., 2014; Perazzolli et al., 2014; Bokulich et al., 2015, 2016a; Burns et al., 2015; Calleja-Cervantes et al., 2015; Piao et al., 2015; Pinto et al., 2015; Zarraonaindia et al., 2015; Holland et al., 2016; Marzano et al., 2016; Portillo et al., 2016). Depending on the region sequenced the data might be similar or differ significantly. For instance, in a study comparing the V4 and V5 region Bokulich et al. (2012), found that the regions resulted in a similar bacterial composition with minor variation in the lower taxa; although the V4 region provided greater taxonomic depth for certain *Proteobacteria* and lactic acid bacteria (LAB) species. In contrast, Campanaro et al. (2014), targeted the V3–V4 and V5–V6 regions of the 16S rRNA region and evaluated the bacterial community associated with grape marc after crushing and 30 days “post fermentation”/storage. A total of 89 genera were identified, however only 31 of these were common in both target regions evaluated.

The fungal ITS regions are the most commonly targeted region for fungal diversity estimation. The classification of general fungi and arbuscula mycorrhizae (AMF) has been accomplished by targeting the ITS region (Bokulich et al., 2013a, 2015, 2016a; Setati et al., 2015; Bouffaud et al., 2016; Holland et al., 2016; Kecskeméti et al., 2016; Marzano et al., 2016; Stefanini et al., 2016), D1–D2 regions of the 26S rRNA (Holland et al., 2014; Taylor et al., 2014) and the partial 18S rRNA gene (Lumini et al., 2010; David et al., 2014; Holland et al., 2016; Grangeteau et al., 2017; De Filippis et al., 2017). The AMF populations derived from these different targets, were similar in genera and showed compositional differences in samples evaluated, highlighting them all as suitable target genes for AMF evaluation (Lumini et al., 2010; Bouffaud et al., 2016). Furthermore, Pinto et al. (2014, 2015) targeted both the ITS2 region and D2 domain of the 26S rRNA region for fungal community analysis. The results revealed that the taxonomic depth for the two evaluated regions was considerably similar, however of these only a portion of the observed OTU's were shared between the two regions and that overall the ITS region provided a slightly higher coverage. Bokulich and Mills (2013) moreover, evaluated several ITS primers, and they found that targeting the ITS1 region demonstrates higher levels of taxonomic classification accuracy (species and genus), the smallest difference between Ascomycota and Basidiomycota amplicon lengths, as well as a maximized sequence coverage. Therefore, overall the ITS1 locus appears to be the most promising target, for a complete overview of the microbial populations in ecological studies.

## Bioinformatics and analysis

High throughput sequencing techniques generally generate large amounts of sequence data, and the only viable option to handle such information, is via automated approaches. There are currently several open source pipelines accessible for overseeing, almost the complete analysis procedure for NGS data. These include MOTHUR, quantitative insights into microbial ecology (QIIME; Kõljalg et al., 2013), metagenomics rapid annotation using subsystem technology (MG-RAST), server and rapid analysis of multiple metagenomes with clustering and annotation pipeline (RAMMCAP; Wooley et al., 2010). These pipelines provide the tools for basic data analysis steps such as data cleaning, sequence clustering, functional annotation, and taxonomic assignments (Kõljalg et al., 2013).

The current section will provide brief overview in the procedures used to analyze high-throughput sequencing data in targeted amplicon sequencing for the vineyard and wine associated microbiome, followed by a brief overview of whole-metagenomics sequencing.

## Target/amplicon sequencing

The analysis of amplicon sequencing data typically undergoes three basic steps; (i) Quality trimming and de-noising; (ii) OTU-picking/clustering, and (iii) taxonomic assignment. Quality-trimming is an essential step used to eradicate erroneous reads obtained through PCR, sequencing instruments and the chemistries behind the sequencing reactions (Bokulich et al., 2013a). To minimize the volume of data for annotation, clustering, and OTU-picking is used. During clustering, pairwise comparison of sequencing is performed with a set percentage identity threshold. Subsequently, a single representative of highly similar sequences is chosen and annotated through BLAST or BLAT algorithms. OTUs can be processed through an open-reference or closed-reference OUT-picking approach. Assignment of species or annotation of functional genes is based on percentage similarity to sequences in specific databases such as Greengenes, UNITE, SILVA, NCBI, SWISSPROT etc.

**Figure 1:**
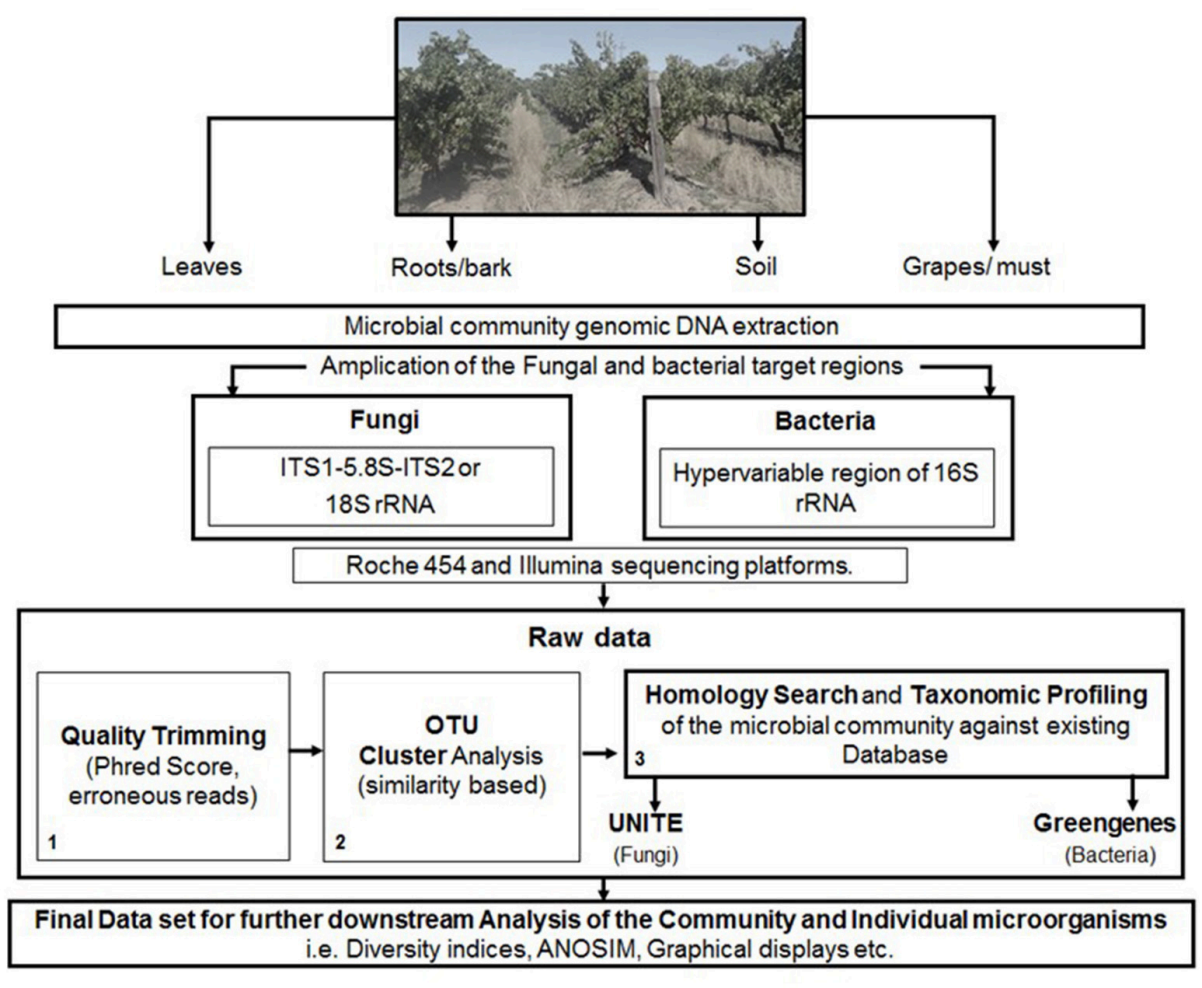
**A schematic representation of the steps involved in targeted amplicon sequencing**.

The analysis of data derived from pyrosequencing during quality trimming typically involves; the removal of barcodes, adapters, and primers, followed by denoising which is used to correct problems associated specifically with 454 pyrosequencer. These typically include the removal of sequences, with ≥6 homopolymers, ambiguous bases and those not meeting Phred score of (20–30). Furthermore, sequences of min and max length can be removed, depending on the target region and possible chimeric sequences (Figure 1).

The data derived from Illumina sequencing platforms undergoes similar demultiplexing and quality trimming apart from denoising. Reads are typically truncated for ≥3 consecutive bases with a quality <1e^−5^, and removed when containing ambiguous base calls, primer/barcode errors or a phred score of <20–30. Furthermore, for paired-end sequencing, the reads are typically joined after quality trimming prior to OTU picking, with all sequences retained, even those not overlapping (Figure 1).

## Shotgun metagenomics sequencing

While the goal in the analysis of the metagenomic data is to reconstruct all the genomes within the environmental sample, the computational intricacy involved makes it unfeasible. Thus, as an alternative two general types of analyses are performed for reconstruction; (i) assembling the reads into contigs, and performing taxonomic classification and functional assignments; (ii) read-based reconstruction of the taxonomic and functional parts of the metagenome. During the assembly of sequences, several problems could arise; for instance, limitation in computational space (Peršoh, 2015), formation of chimeras as a consequence of similarities amongst genomes of related species and variable abundances of genomes within the sample which could potentially result in partial representation (Scholz et al., 2012; Ravin et al., 2015).

Since a mixture of varying amounts of genomic fragments, from different organisms is the result of contig assembly, taxonomic classification can be complicated. Nevertheless, clustering based on the nucleotide composition and coverage carried out by different techniques could sort/bin metagenomic data based on taxonomic status. The clustering efficacy does however rely on various factors. Furthermore, the taxonomic status of the resulting “bins” of contigs is obtained through the identification of phylogenetic marker genes in the bin which was analyzed (Ravin et al., 2015). Additional algorithms have been proposed as an alternative to the cluster based algorithms (Kriseman et al., 2010).

The annotation of the metagenomic contigs can be done using various command-line pipelines and online annotations services, such as MG-RAST, integrated microbial genomes and microbiomes (IMG-M) and community cyberinfrastructure for advanced microbial ecology research and analysis (CAMERA), which in addition to annotation, are able to conduct taxonomic and functional classification as well as pathway reconstruction (Wooley et al., 2010; Desai et al., 2012; Scholz et al., 2012; Ravin et al., 2015). The dependability of the taxonomic assignment and therefore the corresponding information may be decided from scores on sequence similarity and alignment coverage by quality standards or phylogenetic analyses (Peršoh, 2015).

Monitoring complex microbial communities is essential in food fermentations, in which consortia of microbial communities are naturally involved in the processes, such as fermentation and spoilage (Bokulich et al., 2016b). These technological advances, therefore represent an enormous breakthrough for microbial ecology, because metagenomics and NGS allow for in-depth insights into not only the structure, but the function of the most complex microbial communities in their natural environments (Peršoh, 2015). The following section, will therefore focus on metagenomics and how it has been applied to study the vineyard microbial communities.

## Vineyard microbial communities as derived from targeted SGS

SGS technologies have become the tool of choice in deciphering the vineyard and wine microbiome. Most importantly these tools have been employed in microbial surveys that sought to understand how agronomic practices influence microbial community structures and whether there are grapevine organ-specific microbial signatures. Furthermore, it is increasingly becoming important to understand whether there is geographic microbial signatures that contribute to wine typicity.

## Bacterial communities

Several studies have recently employed high-throughput sequencing to evaluate the bacterial communities associated with the vineyard. The most abundant phyla in vineyard soils and grapevine roots include *Proteobacteria, Bacteriodetes, Acidobacteria, Verrucomicrobia, Planctomycetes, Actinobacteria, Chloroflexi, Gemmatimonatedes*, and *Firmicutes* (Burns et al., 2015; Calleja-Cervantes et al., 2015; Zarraonaindia et al., 2015; Faist et al., 2016). Studies suggest that the soil microbial community composition in vineyards closely resembles that of other agricultural ecosystems and is largely structured with respect to soil properties and viticultural area (Burns et al., 2015). Furthermore, soil amendments such as fertilizer and/or compost applications can alter the relative abundances of bacterial groups (Calleja-Cervantes et al., 2015). High-throughput analysis of the grapevine phyllosphere, flowers and grape berry surface, demonstrated that the bacterial communities were predominated by *Proteobacteria* followed by *Firmicutes, Actinobacteria, Acidobacteria*, and *Bacteroidetes* (Perazzolli et al., 2014; Pinto et al., 2014, 2015; Portillo and Mas, 2016; Portillo et al., 2016). The relative abundances of the groups vary depending on the plant tissue or organ. Dominant taxa include members of the genera *Pseudomonas, Sphingomonas, Frigoribacterium, Curtobacterium, Bacillus, Enterobacter, Acinetobacter, Erwinia, Citrobacter, Pantoea*, and *Methylobacterium* (Bokulich et al., 2014, 2016a; Perazzolli et al., 2014; Pinto et al., 2015; Zarraonaindia et al., 2015; Kecskeméti et al., 2016; Portillo and Mas, 2016; Portillo et al., 2016). In contrast, the endophytic community in grape berries mainly comprise *Ralstonia, Burkholderia, Pseudomonas, Staphylococcus, Mesorhizobium, Propionibacterium, Dyella*, and *Bacillus* species (Campisano et al., 2014). However, it is important to note that the bacterial community structure varies amongst grape cultivars, and is also influenced by agronomic practices (Campisano et al., 2014; Perazzolli et al., 2014; Calleja-Cervantes et al., 2015; Pinto et al., 2015; Kecskeméti et al., 2016). Furthermore, development of diseases can result in establishment of different community structures. For instance, graft unions with crown galls were shown to harbor three bacterial OTUs *viz*. *Agrobacterium vitis, Pseudomonas* sp., and *Enterobacteriaceae* sp., that were most abundant in every season, while the three most abundant OTUs in graft unions without a crown gall differed in every season suggesting that crown galls are colonized by a stable bacterial complex (Faist et al., 2016). In other studies, a higher incidence of acetic acid bacteria (AAB) was shown to develop in positive correlation with the *Botryotinia* sp. on grapevine leaves and in botrytized wine fermentations (Bokulich et al., 2012; Pinto et al., 2015). However, Portillo and Mas (2016) demonstrated that this group of bacteria, specifically *Gluconobacter* spp., can persist at high abundance throughout wine fermentation in non-botrytized Grenache fermenting musts, only declining at the end of alcoholic fermentation. Furthermore, the population of *Gluconobacter* was shown to be highly abundant in organic pied-de-cuve Riesling fermentation compared to the conventional fermentation (Piao et al., 2015). AAB were also shown to dominate in low sulfited, uninoculated wine fermentations, compared to *Lactobacillus* and *Lactobacillaceae* that dominated SO_2_-free uninoculated fermentations (Bokulich et al., 2015). Interestingly a low abundance of LAB is often reported with amplicon sequencing phylogenetic surveys (Bokulich et al., 2012; Pinto et al., 2014, 2015). Most importantly, *Oenococcus oeni* seems to be rarely encountered in grape must except in one study where it was found to be dominant in fermentations of Grenache and Carignan grapes (Portillo et al., 2016). However, several studies show that the levels of this species increase during malolactic fermentation and that in fact it is in most cases the dominant taxa (Marzano et al., 2016; Portillo et al., 2016). Other LAB often encountered include *Lactobacillus, Lactococcus, Leuconostoc*, and *Pediococcus* species (Bokulich et al., 2012, 2014; Piao et al., 2015; Pinto et al., 2015; Portillo et al., 2016).

Overall SGS have made it possible to detect bacterial species often overlooked in culture-based methods and community fingerprinting approaches such as DGGE as it can detect species that represent 0.001–1% of the total population. Furthermore, several novel genera believed to be associated with the wine habitat, including, *Candidatus_Liberibacter, Onus, Wolbachia, Komagateaibacter*, and *Shewanella* were detected (Marzano et al., 2016; Portillo and Mas, 2016). In some cases, these rare taxa including *Methylobacterium, Sphingomonas, Acinetobacter, Pseudomonas, Wolbachia*, and *Paracoccus* could be detected until the end of alcoholic fermentation (Bokulich et al., 2012; Piao et al., 2015; Portillo and Mas, 2016). A closer look at supplementary data from various publications suggests that over 100 species are newly associated with grapevine or wine. However, since only partial sequences are used, most of the taxanomic assignments are generally reliable to genus level. Nevertheless, Table 2 shows a representation of a few species that have been identified in various studies and have been shown to persist from the vineyard environment and throughout wine fermentation. Some of the species e.g., *Methylobacterium populi* and *Sphingomonas pseudosanguinis*, were confirmed to be viable at the end of fermentation (Bokulich et al., 2012) and the populations of these genera were also shown to persist in the winery on non-fermentor surfaces (Bokulich et al., 2013b). Further research into these taxa is, however required to evaluate their possible impact in wine fermentation and/or wine quality.

**Table 2:** **A selection of rare bacterial species detected on grapevine leaves (L), Roots (R), Stems, and Shoots (SS), berry surface (B) and in Soil (So), Grape Marc (GM), as well as in must (M) before fermentation (BF), in the middle (MF) and at the end of the alcoholic fermentation (EF)**.

**Genus**	**Species**	**Source**	**Fermentation stage**	**References**
*Acinetobacter*	*A.baumannii* *A.calcoaceticus* *A. guillouiae* *A. johnsonii* *A. junii* *A. lwoffii* *A. rhizosphaerae*	GM, M, R, So	BF	Burns et al., 2015; Piao et al., 2015; Marzano et al., 2016; Portillo et al., 2016
*Candidatus*	*Ca. Accumulibacter unclassified* *Ca. Blochmannia floridanus* *Ca. Blochmannia pennsylvanicus* *Ca. Carsonella ruddii* *Ca. Desulforudis audaxviator* *Ca. Liberibacter* *Ca. Pelagibacter ubique* *Ca. Phytoplasma yellows* *Ca. Sulcia muelleri* *Ca. Vesicomyosocius okutanii*	M	BF/MF/EF	Marzano et al., 2016; Salvetti et al., 2016
*Chryseobacterium*		B, GM, M, So	BF/MF/EF	Campanaro et al., 2014; Burns et al., 2015; Kecskeméti et al., 2016;
*Halomonas*	*H. desiderata* *H. elongata* *H. phoceae* *H. rifensis*	B, M	BF/MF/EF	Bokulich et al., 2015; Marzano et al., 2016; Salvetti et al., 2016
*Komagataeibacter*	*K. europaeus* *K. hansenii* *K. intermedius* *K. kakiaceti* *K. maltaceti* *K. medellinensis* *K. oboediensis* *K. rhaeticus* *K. saccharivorans* *K. sucrofermentans* *K. xylinus*	M	BF/MF/EF	David et al., 2014; Pinto et al., 2014, 2015; Setati et al., 2015
*Methylobacterium*	*M. adhaesivum* *M. dankookense* *M. extorquens* *M. fujisawaense* *M. longum* *M. mesophilicum* *M. populi* *M. radiotolerans* *M. rhodesianum*	M, R, So	BF/MF/EF	Bokulich et al., 2012; Burns et al., 2015; Piao et al., 2015; Marzano et al., 2016; Portillo et al., 2016
*Ralstonia*	*R. solanacearum*	SS/M	BF	Campisano et al., 2014; Marzano et al., 2016; Salvetti et al., 2016
*Sphingomonas*	*S. aerolata* *S. aquatilis* *S. echinoides* *S. endophytica* *S. insulae* *S. melonis* *S. mucosissima* *S. phyllosphaerae* *S. pseudosanguinis* *S. wittichii* *S. yunnanensis*	B, GM, M, R, So	BF/MF	Bokulich et al., 2012; Campanaro et al., 2014; Burns et al., 2015; Piao et al., 2015; Faist et al., 2016; Kecskeméti et al., 2016; Marzano et al., 2016; Salvetti et al., 2016
*Wolbachia*	*W. endosymbiont*	M	BF/MF	Piao et al., 2015; Kecskeméti et al., 2016; Marzano et al., 2016; Portillo and Mas, 2016; Salvetti et al., 2016

## Fungal communities

The fungal communities associated with grapevine have mainly been investigated in must after crushing. Overall, the fungal populations at a phylum level are very similar and mainly comprise the Ascomycota and the most abundant phylum followed by the Basidiomycota (Bokulich et al., 2014; David et al., 2014; Taylor et al., 2014; Pinto et al., 2015; Setati et al., 2015; Kecskeméti et al., 2016). Other phyla such as the Zygomycota and Chytridiomycota are only present in low abundance. Frequently encountered genera of filamentous fungi include *Aspergillus, Alternaria, Penicillium, Cladosporium, Lewia, Davidiella, Erysiphe, Botrytis* and the yeast-like fungus, *Aureobasidium pullulans*, while the yeast genera include *Hanseniaspora, Issatchenkia, Pichia, Candida, Rhodotorula, Lachancea, Metschnikowia, Cryptococcus, Filobasidiella, Sporobolomyces*, and *Torulaspora* (Bokulich et al., 2014; David et al., 2014; Taylor et al., 2014; Pinto et al., 2015; Setati et al., 2015; Wang et al., 2015; Kecskeméti et al., 2016; De Filippis et al., 2017). Generally, the SGS have revealed more filamentous fungal species than yeast species especially those associated with the grape berry surface (Tables 3, 4). These data suggest that most of the yeast genera and species are cultivable but are often missed in culture-based studies due to their presence in minor concentrations. In contrast, for the filamentous fungi, SGS reveals a diversity of possible rot associated taxa such as *Botrytis elliptica* and *Botrytis fabae*. Further studies could look into investigating the prevalence of these species and their contribution to rot.

**Table 3:** **Filamentous fungi detected on grapevine leaves (L), berry surface (B) and in must (M) before fermentation (BF), in the middle (MF) and at the end of the alcoholic fermentation (EF)**.

**Genus**	**Species**	**Source**	**Stage**	**References**
**FILAMENTOUS FUNGI**				
*Albugo*	*A. laibachii*	B		Kecskeméti et al., 2016
*Ascochyta*	*A. fabae, A. rabiei*	M		Setati et al., 2015
*Botrytis*	*Bot. elliptica Bot. fabae*	B/M		Kecskeméti et al., 2016; Setati et al., 2015
*Cadophora*	*C. luteo-olivacea*	B		Kecskeméti et al., 2016
*Catelunostroma*	*C. protearum*	B		Kecskeméti et al., 2016
*Chloroscypha*	*C. enterochroma*	B		Kecskeméti et al., 2016
*Cladosporium*	*C. cucumerinum* *C. exasperatum* *C. flabelliforme* *C. perangustum*	B/M	BF/MF/EF	Bokulich et al., 2014, 2016a; Taylor et al., 2014; De Filippis et al., 2017; Grangeteau et al., 2017; Kecskeméti et al., 2016; Setati et al., 2015
*Cytospora*	*C. sacculus*	M	BF	Wang et al., 2015
*Didymella*	*D. exitialis D. fabae*	B		Kecskeméti et al., 2016
*Gigaspora*	*G. margarita*	B		Kecskeméti et al., 2016
*Glonium*	*G. pusillum*	B		Kecskeméti et al., 2016
*Haplographium*	*H. catenatum*	B		Kecskeméti et al., 2016
*Holtermannia*	*H. corniformis*	B		Kecskeméti et al., 2016
*Hypholoma*	*H. fasciculare*	B		Kecskeméti et al., 2016
*Kabatiella*	*K. microsticta*	M		Setati et al., 2015
*Mycosphaerella*	*M. milleri*	M		De Filippis et al., 2017
*Pandora*	*P. neoaphidis*	L		Pinto et al., 2014
*Peniosphora*	*P. aurantiaca* *P. incarnate*	B		Kecskeméti et al., 2016
*Piptoporus*	*P. betulinus*	B		Kecskeméti et al., 2016
*Puccinia*	*P. punctiformis*	L/B		Pinto et al., 2014; Kecskeméti et al., 2016
*Sarocladium*	*S. strictum*			Kecskeméti et al., 2016
*Sclerotinia*	*S. subarctica*	B/M	BF	David et al., 2014; Kecskeméti et al., 2016; Salvetti et al., 2016
*Sebacina*	*S. vermifera*	B		Kecskeméti et al., 2016
*Sphaeropsis*	*S. sapinea*	B		Kecskeméti et al., 2016
*Stephanonectaria*	*S. keithii*	B		Kecskeméti et al., 2016
*Sydowia*	*S. polyspora*	B		Kecskeméti et al., 2016
*Veluticeps*	*V. berkeleyi*	B		Kecskeméti et al., 2016
*Vuilleminia*	*V. comedens*	B		Kecskeméti et al., 2016
*Zoophthora*	*Z. radicans*	L		Pinto et al., 2014

**Table 4:** **Yeasts detected on grapevine leaves (L), berry surface (B) and in must (M) before fermentation (BF), in the middle (MF) and at the end of the alcoholic fermentation (EF)**.

**Genus**	**Species**	**Source**	**Fermentation stage**	**References**
*Cryptococcus*	*C. tephrensis* *C. chernovii* *C. stepposus*	L/B/M	BF/MF	Bokulich et al., 2014; David et al., 2014; Taylor et al., 2014; Grangeteau et al., 2017; Kecskeméti et al., 2016; Setati et al., 2015; De Filippis et al., 2017
*Filobasidium*	*F. floriforme*	B		Kecskeméti et al., 2016
*Hanseniaspora*	*H. thailandica*	L/M	BF/MF/EF	Wang et al., 2015
*Rhodotorula*	*R. fujisanensis*	L/M	BF	David et al., 2014; Pinto et al., 2014, 2015; Setati et al., 2015
*Schizosaccharomyces*	*S. japonicus*	M	BF/MF/EF	Pinto et al., 2015
*Sclerostagonospora*	*Scl. opuntiae*	M	BF	Bokulich et al., 2014
*Sporobolomyces*	*S. coprosmae S. oryzicola*	M	BF/MF	David et al., 2014; Setati et al., 2015; De Filippis et al., 2017

Several studies have suggested that the microbial community associated with grapevines exhibit regional differentiation (Bokulich et al., 2014, 2016a,b; Taylor et al., 2014; Pinto et al., 2015; Wang et al., 2015). Such regional distinction has been attributed to the dominance of a few species per region. For instance, Bokulich et al. (2014) demonstrated significant association of *Aspergillus* and *Penicillium* spp. with the Chardonnay in Napa, while *Bacteroides, Actinobacteria, Saccharomycetes*, and *Erysiphe necator* were abundant in Central Coast; and *Botryotinia fuckeliana* and Proteobacteria in Sonoma. Similarly, Pinto et al. (2015) showed that *Lachancea* prevailed in the Alentejo appellation, while *Rhodotorula* and *Botrytinia* dominated in the Estremadura appellation, *Hanseniaspora* and *Ramularia* in Bairrada, *Lachancea* and *Rhodotorula* in Dão, *Rhodotorula* and *Erisyphe* in Douro, and *Rhodotorula* and *Alternaria* in Minho appellation. The fungal diversity associated with grapes is also influenced by agronomic practices. Most importantly, studies have shown that vineyards employing conventional, Integrated Pest management systems, Organic, Biodynamic, and Ecophyto practices harbor different fungal communities (David et al., 2014; Setati et al., 2015; Kecskeméti et al., 2016).

Overall, NGS reveal higher diversity compared to other culture-independent methods such as DGGE and qPCR (David et al., 2014; Wang et al., 2015). Furthermore, these methods have detected minor and rare species that are sometimes overlooked with culture-dependent methods and can detect non-culturable cells at the end of fermentation. For instance, some of the studies show the presence yeast genera such as *Kazachstania, Malassezia, Schizosaccharomyces*, and *Debaryomyces* which are typically at low frequency (David et al., 2014; Pinto et al., 2015; Setati et al., 2015; Grangeteau et al., 2017), while cells of *Hanseniaspora* spp. have been detected at the end of fermentation (Wang et al., 2015). Similar to what has been observed with culture-dependent methods, *S. cerevisiae* is rarely encountered in grape must even with NGS technologies. However, the fungal community in fermenting musts tends to be less diverse toward the end of fermentation and is dominated by *Saccharomyces* spp. In some cases, where strong fermentative yeasts such as *Lachancea, Starmerella*, and *Schizosaccharomyces* were present at high frequency in the initial population, they persist until the end of fermentation (Pinto et al., 2015; Wang et al., 2015; Bokulich et al., 2016a). Such species have also been shown to contribute toward taxonomic discrimination between growing regions. There is also increasing evidence that there are broad taxonomic trends underlying varietal patterns. For instance, Bokulich et al. (2014) found differences in Chardonnay, Cabernet sauvignon, and Zinfandel, while Wang et al. (2015) demonstrated that Grenache and Carignan grapes harbored certain distinct taxa. Most recently, Aglianico and Greco di Tufo were also found to harbor different yeast communities (De Filippis et al., 2017). Current data show that there is conflicting outcomes regarding the relative abundances of yeast species in must depending on the methods employed. Therefore, although microbial surveys using amplicon sequencing can detect all species that are retrieved by culture-based methods, and other culture-independent methods, the quantity of certain species tends to vary. In addition, there can be variation in community composition depending on the rRNA gene target. For instance, in the study by Pinto et al. (2015) both the D2 region and the ITS-5.8S region were targeted, however, only 13.2% of the taxa were common between the two data sets. This highlights an important gap with regard to the completeness of the databases and accuracy with regard to taxonomic assignment especially at a species level. Furthermore, amplicon sequencing data still comprise significant percentages of “unclassified” or unassigned OTUs which suggests that the diversity is still to some extent under-represented. Studies evaluating fungal diversity in the vineyard remain limited. Orgiazzi et al. (2012) reported that the soil ecosystem is dominated by the genera *Penicillium* and *Cryptococcus*, the minor fungal groups are mainly dominated by *Glomeromycota* or *Chytridiomycota*. In contrast, the leaf associated microbiome is dominated by early diverging fungal lineages (*Zygomycota*) such as *Rhizopus* and *Mucor* (Pinto et al., 2014), while AMF specific fungi of the soil and grapevine are dominated by *Glomeromycota* (Lumini et al., 2010; Bouffaud et al., 2016). However, more studies need to be performed in order to confidently elucidate the vineyard and grapevine phyllosphere microbiome.

## Whole-metagenomic sequencing

Recently, Salvetti et al. (2016) employed whole genome sequencing for the first in-depth evaluation of the microbial consortium associated with Corvina berries post withering performed in two different conditions. A total of 25 bacterial phyla were detected, nine of which were common and consisted of *Acidobacteria, Actinobacteria, Cyanobacteria, Firmicutes*, and *Proteobacteria*; the latter was predominant, followed by *Firmicutes, Actinobacteria*, and *Bacteroidetes* as reported by Pinto et al. (2014) and Zarraonaindia et al. (2015), who both employed target metagenomics strategies. The class *Gammaproteobacteria* was dominant, which was further represented by *Pseudomonadaceae* in high abundances in the traditional withering and *Enterobacteriaceae* in accelerated withering. Furthermore, both genera *Carnobacterium* and *Enterococcus* previously identified as grape associated by Pinto et al. (2015) was detected using the whole genome sequencing approach. Also, evaluating the eukaryotic community, they reported that *Ascomycota* was the dominant phylum, more specifically the class *Eurotiomycetes*, specifically genera belonging to *Aspergillus* and *Penicillium*, followed by *Sordariomycetes* and *Dothideomycetes*. However, common yeast such as *Aureobasidium, Cryptococcus, Hanseniaspora, Metschnikowia*, and *Sporobolomyces* which are regularly detected in targeted strategies were not detected.

Beyond providing the inventory of the vineyard, whole metagenomic analysis provides the functional information for the evaluated microbiome. For instance, information regarding defense, amino acid metabolism, transport, transcription and carbohydrate metabolism, potentially allowing a greater comparison to be drawn than the assumed microbial diversity and composition (Campanaro et al., 2014; Salvetti et al., 2016).

## Conclusion

The invaluable contribution of metagenomic approaches in deciphering the vineyard microbiome and its application provides great insights in the microbial community composition and structure of both bacteria and fungi. Metagenomic approaches provide an opportunity to study the entire microbial population and not just one group as typically done with culture-based methods. Consequently, it has been possible to assess the population dynamics during fermentation, to evaluate grapevine disease complexes and unravel unique microbial signatures present in grapevine and not in neighboring plants. Furthermore, these approaches have been valuable in understanding the influence of vineyard management practices on the grapevine microbiome. Based on the existing research papers, it appears as though the grapevine microbiome is less complex compared to other ecosystems such as soil and that a large proportion of the yeast species associated with the grape and wine environment are cultivable. This is advantageous as the species can then be evaluated for potential genes, enzymes etc. that can be of importance for winemaking. However, most of the studies show that a significant percentage of the sequence data (OTU's) remained unassigned. This problem highlights existing challenges with sequence databases used for taxonomic assignment that are not complete and for this technology to be furthered in future means that the expansion of the databases are crucial. Nevertheless, based on existing data, sequence-based methods reveal similar fungal species compared to culture-dependent methods, especially regarding the yeasts which are relevant in wine fermentation. The discovery of new species associated with the grape and wine microbiome holds tremendous potential to mine them for novel properties that would improve wine fermentation, aroma and style.

## Author contributions

HM wrote the first draft of the review; MdT and MS proofed the drafts and finalized the review.

## Funding

This work was funded by the National Research Foundation (NRF) [grant number 101998] and Winetech SU IWBT 16/02. Opinions expressed and conclusions arrived at are those of the authors and are not necessarily to be attributed to the funding agencies.

### Conflict of interest statement

The authors declare that the research was conducted in the absence of any commercial or financial relationships that could be construed as a potential conflict of interest.
